# Detection of Respiratory Pathogens in Saliva and Mouthwash Samples in Children

**DOI:** 10.1093/jpids/piaf105

**Published:** 2025-11-22

**Authors:** Ville Lindholm, Suvi Mattila, Kimmo Halt, Niko Paalanne, Tytti Pokka, Vesa Mäki-Koivisto, Laura E Savolainen, Minna Honkila, Terhi Ruuska-Loewald

**Affiliations:** Research Unit of Clinical Medicine and Medical Research Center Oulu (MRC Oulu), University of Oulu, Oulu, Finland; Department of Pediatrics and Adolescent Medicine, Oulu University Hospital, Oulu, Finland; Research Unit of Clinical Medicine and Medical Research Center Oulu (MRC Oulu), University of Oulu, Oulu, Finland; Department of Pediatrics and Adolescent Medicine, Oulu University Hospital, Oulu, Finland; Research Unit of Clinical Medicine and Medical Research Center Oulu (MRC Oulu), University of Oulu, Oulu, Finland; Department of Pediatrics and Adolescent Medicine, Oulu University Hospital, Oulu, Finland; Research Unit of Clinical Medicine and Medical Research Center Oulu (MRC Oulu), University of Oulu, Oulu, Finland; Department of Pediatrics and Adolescent Medicine, Oulu University Hospital, Oulu, Finland; Research Unit of Clinical Medicine and Medical Research Center Oulu (MRC Oulu), University of Oulu, Oulu, Finland; Research Service Unit, Oulu University Hospital, Oulu, Finland; NordLab, Oulu, Finland; NordLab, Oulu, Finland; Research Unit of Clinical Medicine and Medical Research Center Oulu (MRC Oulu), University of Oulu, Oulu, Finland; Department of Pediatrics and Adolescent Medicine, Oulu University Hospital, Oulu, Finland; Research Unit of Clinical Medicine and Medical Research Center Oulu (MRC Oulu), University of Oulu, Oulu, Finland; Department of Pediatrics and Adolescent Medicine, Oulu University Hospital, Oulu, Finland; Biocenter Oulu, University of Oulu, Oulu, Finland

**Keywords:** saliva samples, respiratory pathogens, multiplex polymerase chain reaction panel

## Abstract

**Background:**

Less invasive alternatives for nasopharyngeal swab samples are needed for the detection of respiratory pathogens in children.

**Methods:**

Prospective diagnostic study comparing the accuracy and convenience of saliva and mouthwash samples with nasopharyngeal swab samples in detecting respiratory pathogens using a multiplex polymerase chain reaction panel for 19 respiratory viruses or viral subtypes and 4 respiratory bacteria. Acutely ill children aged 2–16 years with a suspected respiratory tract infection provided saliva and mouthwash samples in addition to a nasopharyngeal swab sample.

**Results:**

Altogether, 850 samples were obtained from 302 children, including 300 nasopharyngeal swab, 277 saliva, and 273 mouthwash samples. Among 302 participants (mean age, 9.7 years [SD, 3.0], 151 males [50%]), at least 1 respiratory pathogen was detected in 219 (73%). The most common pathogens in nasopharyngeal swab samples were rhinovirus or enterovirus (86 [29%]), *Mycoplasma pneumoniae* (46 [15%]), influenza viruses A and B (32 [11%]), adenovirus (15 [5.0%]), and respiratory syncytial virus (14 [4.7%]). The sensitivity of saliva samples was 71% (95% CI, 60%-81%) for rhinovirus or enterovirus, 93% (95% CI, 82%-99%) for *M. pneumoniae*, 86% (95% CI, 64%-97%) for influenza A virus, and 88% (95% CI, 47%-100%) for influenza B virus. The sensitivity of mouthwash samples was 48% (95% CI, 36–60%) for rhinovirus or enterovirus, 90% (95% CI, 77%-97%) for *M. pneumoniae*, 73% (95% CI, 50%-89%) for influenza A virus, and 67% (95% CI, 30%-93%) for influenza B virus. In total, 99% (257/260) of the children preferred either saliva or mouthwash samples to nasopharyngeal swab samples.

**Conclusions:**

The sensitivity of saliva and mouthwash samples varied across respiratory pathogens in children. Less invasive saliva samples may serve as an alternative to nasopharyngeal swab samples for detecting certain respiratory pathogens in children.

## INTRODUCTION

Multiplex polymerase chain reaction (PCR) panels can detect multiple respiratory pathogens simultaneously. In randomized controlled trials, the clinical benefit of active testing for all respiratory pathogens in acutely ill children has been limited.^1^^,^^2^ Yet, testing for influenza, atypical respiratory bacteria, and *Bordetella pertussis* is considered beneficial in children. For influenza, randomized controlled trials have shown that testing reduces further testing, antibacterial prescriptions, and length of stay, while increasing effective antiviral prescriptions in emergency departments (ED).^3–7^ Testing for *B. pertussis*^8^^,^^9^ and *Mycoplasma pneumoniae*^10^^,^^11^ is recommended for patients with suspected infections caused by these pathogens, as targeted antibiotic therapies against them are available.

Nasopharyngeal swabs (NPSs), the most common respiratory samples,^12^ are invasive and inconvenient.^13^^,^^14^ Accordingly, there is a clinical need for less invasive sampling methods, especially in children. During the COVID-19 pandemic, saliva samples were used for both adults^15^ and children^16–18^ to detect severe acute respiratory syndrome coronavirus 2 (SARS-CoV-2). Two small studies have previously compared the diagnostic accuracy of saliva samples to that of NPS samples analyzed by multiplex PCR in pediatric patients.^19^^,^^20^ In a study of 57 children,^19^ any respiratory pathogen was detected in 76% of saliva samples and in 93% of nasopharyngeal samples, with rhinovirus as the most prevalent finding. Another study of 83 children^20^ reported a 49% positive detection rate for saliva-collecting sponge samples in children with a positive nasopharyngeal sample. Thus, there is limited data on the accuracy of saliva and mouthwash samples in detecting respiratory pathogens using multiplex PCR in children.

In this prospective diagnostic study using parallel testing in 302 children, we set out to investigate whether saliva and mouthwash samples could be used as alternatives to NPS samples for detecting respiratory pathogens in acutely ill children.

## METHODS

### Study Design and Oversight

This observational, prospective diagnostic study compared the accuracy of saliva and mouthwash samples with NPS samples in detecting respiratory pathogens in acutely ill children and adolescents under 16 years of age. The testing of 3 sample types was performed in parallel using a multiplex PCR panel for respiratory pathogens. The study was an investigator-driven, single-center study at the Pediatric Emergency Department of Oulu University Hospital, Finland, from March 22, 2022, to November 1, 2024. During the study, the Pediatric ED served both as a walk-in and a referral clinic, with a total of 12 000 annual visits. The hospital paid for the testing of NPS samples obtained for clinical indications, and the study group paid for the parallel testing of saliva and mouthwash samples. The diagnostic study protocol was reviewed by the regional ethics committee at Oulu University Hospital, Finland (EETTMK: 95/2021). Written informed consent was obtained from all participants over 6 years of age and all legal guardians of participants under 16 years of age. This study followed the Standards for Reporting of Diagnostic Accuracy reporting guidelines.

### Study Population

Acutely ill children aged 2 to 16 years were eligible to participate if they had a suspected respiratory tract infection and a clinical indication for obtaining an NPS sample. Patients requiring immediate cardiopulmonary resuscitation or transfer to the intensive care unit, as well as families unable to provide informed consent in Finnish, were excluded. The study nurse offered participation to a consecutive series of eligible patients during office hours and enrolled the participants after obtaining written informed consent. Additionally, ED nurses could offer participation to eligible participants at other times. Families and participants reported the onset of preceding symptoms and recent eating and drinking. The study physicians reviewed the electronic medical records of the study subjects.

### Samples and Testing

Parallel testing of NPS, saliva, and mouthwash samples was performed. The nurses collected NPS samples, based on a clinical indication, by passing a flocked swab (FLOQSwabs; Copan Diagnostics, Inc., CA, U.S.) through a nostril, rotating it on the nasopharynx, and placing the swab directly into the testing cartridge or the 3.0 mL Universal Transport Medium (Copan Diagnostics, Inc., CA, U.S.). Saliva samples were collected using the PAXgene Saliva Collector (PreAnalytiX GmbH, Hombrechtikon, Switzerland).^21^ The participants spat saliva up to the 2.0 mL line, indicating sufficient volume. When the funnel of the saliva collector was removed, 1.0 mL of stabilization solution was released into the tube. After closing the tube, it was inverted at least 5 times. Mouthwash samples were collected by asking participants to take 5 mL of normal saline (0.9%) in the oropharynx for 15 s, gargle the saline if they could, and then spit the saline into a sterile Vacutest urine container (Vacutest Kima, Arzergrande, Italy). The mouthwash sample was always collected last. A 0.3 mL aliquot of each sample was drawn by a pipette and transferred into the testing cartridge, as instructed by the manufacturer.

All parallel samples were tested using a multiplex PCR panel (QIAstat-Dx Respiratory SARS-CoV-2 Panel, Qiagen, Hilden, Germany). The results of all 3 tests were immediately available to the physician in the ED. The panel included 19 viruses or viral subtypes (adenovirus, bocavirus, coronaviruses 229E, HKU1, NL63, OC43, and SARS-CoV-2, human metapneumovirus A/B, influenza A and B viruses, influenza A virus subtypes H1N1/2009, H1 and H3, parainfluenza viruses 1 to 4, respiratory syncytial virus A/B, and rhinovirus/enterovirus) and 4 bacteria (*B. pertussis*, *Legionella pneumophila*, *M. pneumoniae*, and *Chlamydia pneumoniae)*. The panel did not differentiate between rhinoviruses and enteroviruses. *C. pneumoniae* was included in the multiplex PCR panel in October 2023. In addition to the test results (positive or negative), cycle threshold (CT) values, which inversely correlate with pathogen load, were obtained.

### Convenience of Sampling

Participants and their guardians recorded the duration of the sample collection (minutes). The participants reported the ease of providing different samples using a visual analogue scale (VAS) ranging from 0 to 10.

### Analysis

#### Sample Size

We assumed the prevalence of any positive finding to be 70% and the sensitivity of NPS samples to be 90%. We considered saliva or mouthwash samples to be a clinically usable alternative if their sensitivity was 80%. With an α error of 5% and a statistical power of 80%, a sample size of 153 participants was required for the study.^22^ The study was continued after the minimum sample size was reached to include participants with a respiratory bacterial infection because there were ongoing epidemics of both *M. pneumoniae* and *B. pertussis* infections in Finland in 2024.

#### Statistical Analysis

**Figure 1:**
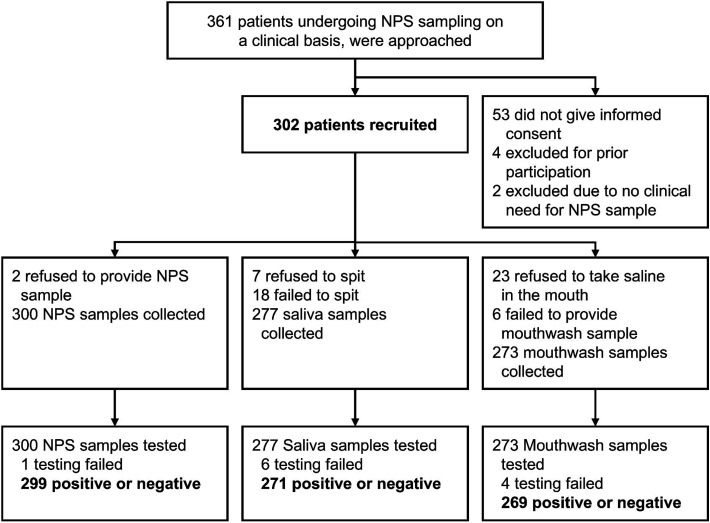
Study Design. The Study Included All Samples that Yielded a Positive or Negative Result (299 Nasopharyngeal Swab, 271 Saliva, and 269 Mouthwash Samples). For Diagnostic Accuracy Analysis, Against NPS as a Clinical Reference Standard, Paired NPS and Saliva Samples from 268 Participants, and Paired NPS and Mouthwash Samples from 267 Participants were Used. The Samples, in which Testing Failed, were Considered Negative. Abbreviations: NPS, nasopharyngeal swab.

All samples with valid positive or negative results were included in the analysis (Figure 1). The reasons for testing failure and unobtained samples were reported (Table 1). The sensitivity and specificity of saliva and mouthwash samples for each pathogens were calculated using NPS sample results as the gold standard. The number of co-detections of different sample types was reported.

**Table 1:** Baseline Demographics and Clinical Characteristics of the Participants

**Characteristic**	**n = 302**
**Age range**, y	2.0–16.3
Mean age, y (SD)	9.7 (3.0)
**Male**, n (%)	151 (50)
**Underlying medical condition**, n (%)	121 (40)
Allergy or atopy, n (%)	44 (15)
Asthma, n (%)	29 (10)
Other, n (%)	66 (22)
**Regular medication**, n (%)	85 (28)
Inhaled corticosteroid, n (%)	34 (11)
Other, n (%)	71 (24)
**Symptoms**	
Median duration, d (IQR)	4 (2–8)
Duration range, d	0–45
Fever, n (%); median duration, d (IQR)	262 (87); 3 (2–6)
Cough, n (%); median duration, d (IQR)	216 (72); 5 (3–10)
Rhinitis, n (%); median duration, d (IQR)	149 (50); 4 (2–6)
Respiratory distress or wheezing, n (%); median duration, d (IQR)	111 (38); 2 (1–4)
Vomiting, n (%); median duration, d (IQR)	93 (31); 1 (1–2)
Abdominal pain, n (%); median duration, d (IQR)	91 (30); 2 (1–4)
Diarrhea, n (%); median duration, d (IQR)	42 (14); 2 (1–4)
Conjunctivitis, n (%); median duration, d (IQR)	34 (11); 2 (1–3)
**Antimicrobial treatment**, n (%)	164 (54)
**Hospital admission**, n (%); median duration, d (IQR)	147 (49); 1.5 (0.9–2.2)
**Diagnosis at discharge**	
RTI, n (%)	179 (59)
Pneumonia, n (%)	59 (20)
Bronchiolitis, n (%)	30 (10)
Lower RTI, other/NAS, n (%)	6 (2)
Upper RTI, n (%)	65 (22)
RTI, other/NAS, n (%)	19 (6)
Infectious disease, other/NAS, n (%)	49 (16)
Other than infectious disease, n (%)	32 (11)
Symptom diagnosis / no diagnosis, n (%)	42 (14)

Abbreviations: RTI, respiratory tract infection; NAS, not able to specify

Sensitivity and specificity were reported as proportions with 95% confidence intervals (CI) calculated using the Clopper–Pearson method. Mean CT values were reported for all pathogens. CT values were compared pairwise between sample types using a paired *t*-test. Opinions on the ease of sampling methods were compared using Wilcoxon’s test. IBM SPSS Statistics software for Windows version 26.0.0.1 (International Business Machines Corporation, U.S.) and StatsDirect statistical software version 3.3.6 (StatsDirect Ltd., England) were used for the statistical analyses.

## RESULTS

### Study Population and Samples

A total of 302 participants with suspected respiratory infections were recruited (Figure 1). The mean age of the study subjects was 9.7 years (SD, 3.0; range, 2.0–16.3 years) (Table 1). The median duration of the preceding symptoms was 4 days (IQR 2–8; range 0–45). In total, 850 samples were obtained from 302 children, including 300 NPS, 277 saliva, and 273 mouthwash samples (Table S1). Testing failed in 1 NPS, 6 saliva, and 4 mouthwash samples (Figure S1). Altogether, 268 participants (89%) had both NPS and saliva sample test results available, and 267 participants (88%) had both NPS and mouthwash sample test results available. 247 participants (82%) had results available for all 3 sample types.

### Detection of Respiratory Pathogens in Different Sample Types

At least, 1 respiratory pathogen in any sample was detected in 219 participants (73%). Of these, 181 had a single pathogen detection, 31 (10%) had 2 pathogens detected simultaneously, and 7 (2%) had co-detection of 3 or 4 pathogens (Table S2).

*M. pneumoniae* was detected in 46 (15%) NPS, 60 (22%) saliva, and 55 (20%) mouthwash samples. *B. pertussis* was detected in 4 (1.3%) NPS, 5 (1.8%) saliva, and 4 (1.5%) mouthwash samples. *C. pneumoniae* was detected in 1 (0.4%) saliva sample. No *L. pneumophila* was detected. Rhinovirus or enterovirus was detected in 86 (29%) NPS, 59 (22%) saliva, and 38 (14%) mouthwash samples. Influenza A virus was detected in 23 (7.7%) NPS, 19 (7.0%) saliva, and 17 (6.3%) mouthwash samples. Influenza B virus was detected in 9 (3.0%) NPS, 7 (2.6%) saliva, and 6 (2.2%) mouthwash samples. Other respiratory viruses were also detected in all sample types (Supplementary Table S3).

### Diagnostic Accuracy of Saliva Samples against NPS Samples

For bacterial detection, the sensitivity of saliva samples was 93% (95% CI, 82%-99%) for *M. pneumoniae* and 75% (95% CI, 19%-99%) for *B. pertussis*. Specificity was 92% (95% CI, 88%-95%) for *M. pneumoniae* and 97% (95% CI, 94%-99%) for *B. pertussis* (Table 2).

**Table 2:** Diagnostic Accuracy of Saliva Samples (*n* = 268) and Mouthwash Samples *(n* = 267) Against Paired NPS Samples

	**Saliva**		**Mouthwash**	
	**Sensitivity** (95% CI)	**Specificity** (95% CI)	**Sensitivity** (95% CI)	**Specificity** (95% CI)
*Mycoplasma pneumoniae*	93 (82–99) 42/45	92 (88–95) 205/223	90 (77–97) 38/42	92 (88–96) 208/22*5*
*Bordetella pertussis*	75 (19–99) 3/4	97 (94–99) 262/270	67 (9–99) 2/3	99 (97–100) 262/264
*Chlamydia pneumoniae*	NA 0/0	99 (97–100) 180/181	NA 0/0	100 (98–100) 179/179
Rhinovirus or enterovirus	71 (60–81) 54/76	98 (96–100) 189/192	48 (36–60) 36/75	98 (96–100) 189/192
Influenza A virus	86 (64–97) 18/21	100 (99–100) 247/247	73 (50–89) 16/22	100 (99–100) 245/245
Influenza B virus	88 (47–100) 7/8	100 (99–100) 260/260	67 (30–93) 6/9	100 (99–100) 258/258
Adenovirus	71 (42–92) 10/14	98 (95–99) 248/254	62 (32–86) 8/13	99 (97–100) 252/254
Respiratory syncytial virus	83 (52–98) 10/12	100 (99–100) 256/256	62 (32–86) 8/13	100 (99–100) 254/254
Parainfluenza viruses 1–4	71 (29–96) 5/7	100 (98–100) 260/261	43 (10–82) 3/7	100 (99–100) 260/260
Human metapneumovirus	100 (48–100) 5/5	100 (98–100) 262/263	83 (36–100) 5/6	99 (97–100) 259/261
Coronavirus^a^	50 (12–88) 3/6	100 (99–100) 262/262	50 (12–88) 3/6	100 (99–100) 261/261
SARS-CoV-2	60 (15–95) 3/5	100 (99–100) 263/263	25 (1–81) 1/4	100 (99–100) 263/263
Bocavirus	0 (NA) 0/2	100 (98–100) 265/266	0 (NA) 0/2	100 (99–100) 265/265

Sensitivity and specificity are reported as proportions with 95% confidence intervals (CI) calculated using the Clopper–Pearson method.

a) Coronavirus included detections in any tested coronaviruses, except SARS-CoV-2: HKU1 (*n* = 2), OC43 (*n* = 2), 229E (*n* = 1), or NL63 (*n* = 1).

Abbreviations: NPS, nasopharyngeal swab; NA, not applicable.

For the most common viruses, the sensitivity of saliva samples was 71% (95% CI, 60%-81%) for rhinovirus or enterovirus, 86% (95% CI, 64%-97%) for influenza A virus, 88% (95% CI, 47%-100%) for influenza B virus, 71% (95% CI, 42%-92%) for adenovirus, 83% (95% CI, 52%-98%) for respiratory syncytial virus, and 71% (95% CI, 29%-96%) for parainfluenza viruses 1–4. Specificity was 98%-100% for all viruses (Table 2).

### Diagnostic Accuracy of Mouthwash Samples against NPS Samples

For bacterial detection, the sensitivity of mouthwash samples was 90% (95% CI, 77%-97%) for *M. pneumoniae* and 67% (95% CI, 9%-99%) for *B. pertussis*. Specificity was 92% (95% CI, 88%-96%) for *M. pneumoniae* and 99% (95% CI, 97%-100%) for *B. pertussis* (Table 2).

For the most common viruses, the sensitivity of mouthwash samples was 48% (95% CI, 36%-60%) for rhinovirus or enterovirus, 73% (95% CI, 50%-89%) for influenza A virus, 67% (95% CI, 30%-93%) for influenza B virus, 62% (95% CI, 32%-86%) for adenovirus, 62% (95% CI, 32%-86%) for respiratory syncytial virus, and 43% (95% CI, 10%-82%) for parainfluenza viruses 1– 4. Specificity was 98%-100% for all viruses (Table 2).

### Cycle Threshold Values

CT values, which inversely correlate with pathogen load, were lower for most viral pathogens in NPS samples than in saliva and mouthwash samples. CT values were higher for *M. pneumoniae* in NPS samples than in saliva samples (mean difference, 2.0; SD 6.2; *P*=.049) but lower than in mouthwash samples (mean difference, 2.6; SD 5.9; *P*=.011) (Figures S2 through S3).

### Collection Time and the Ease of Sampling

**Figure 2:**
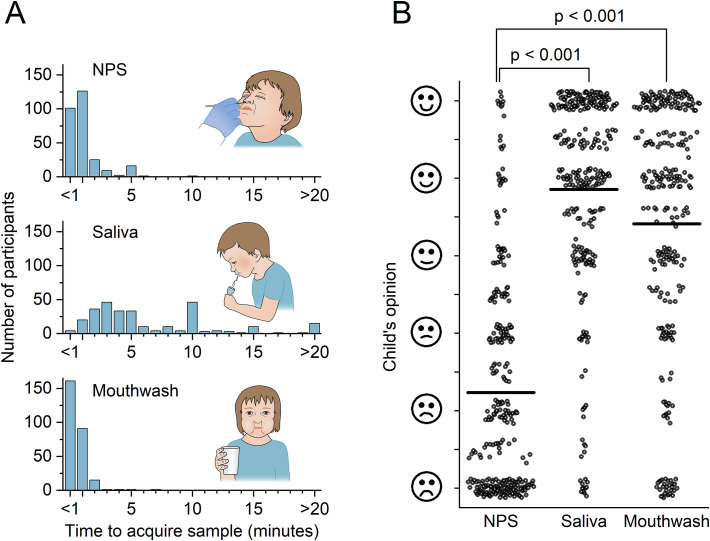
The Ease of Sampling and the Time Required to Obtain Samples. A) The Time Required to Obtain Samples. B) Children’s Opinions About Sampling were Obtained Using a Visual Analogue Scale. Answers were Provided by 295 Children for NPS, 293 Children for Saliva, and 283 Children for Mouthwash Samples. Vertical Lines Indicate the Mean Values. Sampling Methods were Compared using Wilcoxon’s Test.

The median collection time was 1 min (IQR 0.5–1) for NPS, 5 min (IQR 3-10) for saliva, and 0.3 min (IQR 0.2–1) for mouthwash samples (Figure 2 A). A total of 172 children provided a saliva sample within 5 min (61%) and 246 within 10 min (87%).

After sampling, 154 children (59%) preferred saliva samples, 103 (40%) preferred mouthwash samples, and 3 (1.2%) preferred NPS sampling. Twenty-nine participants chose multiple sampling methods, while 13 did not provide an opinion. Based on the VAS score, the children considered the saliva sample the most convenient to provide, followed by the mouthwash and NPS samples (Figure 2 B).

## DISCUSSION

This diagnostic study of 302 children compared saliva and mouthwash samples to NPS samples in detecting respiratory pathogens. The sensitivity of saliva samples varied across respiratory pathogens, being highest for influenza viruses (86-88%) and *M. pneumoniae* (93%). Mouthwash samples had low sensitivity for respiratory viruses. Children preferred saliva and mouthwash samples over NPS samples. Less invasive saliva samples may serve as an alternative to nasopharyngeal swab samples in detecting certain respiratory pathogens in children.

Even though children often find nasopharyngeal swab samples unpleasant, the number of children has been limited in previous studies assessing saliva samples for the detection of respiratory pathogens,^19^^,^^20^^,^^23^ except for the detection of SARS-CoV-2 (Table S4). Our present findings are in line with previous studies on adults. During the influenza season, in a diagnostic study of 469 adult patients, the sensitivity of NPS samples was 86% as compared to 83% of saliva samples for detecting any respiratory pathogen with a multiplex PCR panel.^24^ In another study of 236 adult patients, the proportion of positive samples using a multiplex PCR panel was 78% for NPS and 76% for saliva samples.^25^

Respiratory pathogens with available targeted antimicrobial therapy are the most relevant to detect in patients with respiratory infections. In the present study, the number of patients with *M. pneumoniae* and *B. pertussis* was high, primarily due to a large outbreak of *M. pneumoniae*.^26^ To our knowledge, our study is the first with a sufficient sample size to demonstrate that saliva is a sensitive specimen material for the detection of *M. pneumoniae.*^24^ This finding seems logical, as oropharyngeal swab samples may be better at detecting *M. pneumoniae* compared to NPS samples.^27–29^

For respiratory viruses with the available targeted antimicrobial therapy, saliva samples detected 86% of the children with influenza in this study. A study that included 265 influenza patients found a similar sensitivity of saliva (90% for influenza A and 91% for influenza B) and NPS samples (90% for influenza A and 89% influenza B), but the number of children was not reported.^24^ Another study of 385 adults with flu-like symptoms found that saliva samples were sensitive in detecting influenza.^30^

In our study, mouthwash samples were faster to provide but less sensitive than saliva samples in detecting respiratory pathogens. Possible explanations include an insufficient amount of pathogen nucleic acid in the mouthwash sample, dilution, the absence of stabilization solution, or the presence of inhibitory factors.^31^ We chose to use saline for the mouthwash instead of water,^32^ which would have had a more neutral taste to children.

The main strength of the study is the clinically relevant study idea that diagnostics of respiratory pathogens could be performed in children and adolescents in a more convenient and child-friendly way using saliva samples than obtaining unpleasant nasopharyngeal swab samples. We obtained more than 800 samples from 302 children and tested them in parallel. Thus, the sample size was sufficient to give a reliable estimate of sensitivity combined for all respiratory pathogens and separately for the main pathogens with the available targeted antimicrobial therapy. Our findings could result in changes in clinical practice in health-care units performing testing for respiratory pathogens in children and adolescents.

### Limitations

This study has some limitations. Our study results are generalizable to only children and adolescents who can provide saliva samples to saliva collection tubes. Lower sensitivities of saliva samples have been observed in younger children, particularly in those with small amounts of saliva available, often collected with sponges.^19^^,^^20^ We did not assess whether a smaller amount of saliva, which could ease sampling, would have been sufficient. We could not distinguish viral shredding, bacterial colonization, and persistent carriage after acute infection, even though we included only children with a suspected respiratory infection. We chose to use the commonly used NPS samples as the clinical reference standard, even though they may be imperfect in detecting some pathogens. NPS samples appear to have low sensitivity for some pathogens, such as *M. pneumoniae*, ^27^ and low specificity for others, such as rhinovirus.^33^ Imperfect clinical reference standard, i.e., commonly used NPS in this case, may influence sensitivity and specificity estimates of the tests under evaluation.^34^ Finally, we did not use singleplex PCR in the analysis as we compared the diagnostic accuracy of multiplex PCR panel between specimen types.

## CONCLUSIONS

Saliva samples may serve as a more child-friendly alternative to NPS samples for detecting certain respiratory pathogens in children and adolescents.

## Abbreviations

PCR, polymerase chain reaction; ED, emergency department; NPS, nasopharyngeal swab; CT, cycle threshold; VAS, visual analogue scale; RTI, respiratory tract infection.

## Supplementary Material

Supplemental_material_piaf105

## Data Availability

Deidentified individual participant data (including data dictionaries) will be made available upon publication to researchers who provide a methodologically sound proposal for use in achieving the goals of the approved proposal. Proposals should be submitted to terhi.ruuska-loewald@oulu.fi.
