# Supplementary material for: Detection of Respiratory Pathogens in Saliva and Mouthwash Samples in Children
Source: J Pediatric Infect Dis Soc. 2025 Nov 22;14(12):piaf105. doi: 10.1093/jpids/piaf105 (PMC12771362; doi:10.1093/jpids/piaf105)
Supplement: Supplemental_material_piaf105 [file Supplemental_material_piaf105.docx]

**Supplemental material**

**Table of Contents**

2 Supplementary Table 1. Circumstances before sample collection

3 Supplementary Table 2. Co-detections

4 Supplementary Table 3. Number of pathogens detected by different sample types

5 Supplementary Figure 1: The samples and results by age

6 Supplementary Figure 2. Cycle threshold values of all samples

7 Supplementary Figure 3. Pairwise comparisons of cycle threshold values

8 Systematic review

10 Supplementary Figure 4. Studies screened for the systematic review

11 Supplementary Table 4. Studies included in the systematic review

14 References

**Supplementary Table 1.** **Circumstances before sample collection**

| **Recent ingestions** | **< 0.5 h** | | **0.5–1 h** | | **> 1 h** | |
| --- | --- | --- | --- | --- | --- | --- |
| Any food, n (%) | 16 (5) | | 53 (18) | | 230 (76) | |
| Chewing gum, n (%) | 4 (1) | | 9 (3) | | 284 (94) | |
| Water, n (%) | 34 (11) | | 82 (27) | | 184 (61) | |
| Any drink other than water, n (%) | 32 (11) | | 105 (35) | | 164 (54) | |
| **< 2.0 mL of saliva sample**, n (%) | 18 (6) | | | | | |
| **Gargling instead of mouthwash**, n (%) | 81 (27) | | | | | |
| **Timing of study samples compared to NPS**, cumulative n (%) | < 3 h | < 16 h | | < 24 h | | < 48 h |
|  | 210 (70) | 266 (89) | | 290 (97) | | 299 (99)^a^ |

^a^One participant had samples acquired 3 days after NPS (3 days). All 3 samples were negative.

**Supplementary Table 2. Co-detections**

| **Case** | **Detections (Pathogen [samples detected in])** | | | |
| --- | --- | --- | --- | --- |
| 1 | RV/EV (N,S) | AdV (N) | PIN3 (N) | BoV (N) |
| 2 | RV/EV (N) | MP (All) | AdV (N) |  |
| 3 | RV/EV (All) | MP (N,S) | AdV (N) |  |
| 4 | RV/EV (N) | MP (All) | BoV (N) |  |
| 5 | RV/EV (All) | CoV-NL63 (N) | RSV (N,S) |  |
| 6 | RV/EV (N,S) | CoV-OC43 (All) | hMPV (M) |  |
| 7 | RSV (All) | CoV-HKU1 (N,S) | SARS-CoV-2 (N) |  |
| 8 | RV/EV (N,S) | MP (All) |  |  |
| 9 | RV/EV (N,S) | MP (All) |  |  |
| 10 | RV/EV (N) | MP (All) |  |  |
| 11 | RV/EV (S) | MP (S,M) |  |  |
| 12 | RV/EV (N) | MP (S,M) |  |  |
| 13 | RV/EV (N) | MP (All) |  |  |
| 14 | RV/EV (N) | MP (N) |  |  |
| 15 | RV/EV (N) | MP (All) |  |  |
| 16 | RV/EV (N) | MP (N) |  |  |
| 17 | RV/EV (N) | MP (N,S) |  |  |
| 18 | RV/EV (N,S) | AdV (S) |  |  |
| 19 | RV/EV (N) | AdV (N,S) |  |  |
| 20 | RV/EV (All) | PIN3 (N,S) |  |  |
| 21 | RV/EV (All) | PIN3 (N) |  |  |
| 22 | RV/EV (N,S) | BoV (S) |  |  |
| 23 | RV/EV (M) | RSV (N) |  |  |
| 24 | RV/EV (N,M) | hMPV (N) |  |  |
| 25 | MP (S) | AdV (All) |  |  |
| 26 | MP (S,M) | AdV (S,M) |  |  |
| 27 | MP (S,M) | AdV (All) |  |  |
| 28 | MP (All) | AdV (All) |  |  |
| 29 | MP (S,M) | CoV-229E (N) |  |  |
| 30 | MP (All) | SARS-CoV-2 (N,S) |  |  |
| 31 | AdV (N,S) | BoPe (All) |  |  |
| 32 | AdV (N) | InfA-H09 (N) |  |  |
| 33 | AdV (S) | CP (S) |  |  |
| 34 | RSV (N) | CoV-HKU1 (All) |  |  |
| 35 | RSV (All) | SARS-CoV-2 (N) |  |  |
| 36 | InfB (All) | BoPe (N,S) |  |  |
| 37 | InfB (All) | PIN4 (S) |  |  |
| 38 | BoV (N) | PIN4 (N) |  |  |

Abbreviations N, nasopharyngeal swab sample; S, saliva sample; M, mouthwash sample; RV/EV, rhinovirus or enterovirus; AdV, adenovirus; PIN, parainfluenza virus; BoV, bocavirus; MP, *Mycoplasma pneumoniae*; CoV, coronavirus; RSV, respiratory syncytial virus; hMPV, human metapneumovirus; BoPe, (*Bordetella pertussis*; InfA, influenza A virus; CP, *Chlamydia pneumoniae*.

**Supplementary Table 3. Number of pathogens detected by different sample types**

|  | **Any**  **sample** | **NPS**  **sample** | **Saliva**  **sample** | **Mouthwash**  **sample** |
| --- | --- | --- | --- | --- |
| ***Mycoplasma pneumoniae*** | **65** | **46** | **60** | **55** |
| ***Bordetella pertussis*** | **6** | **4** | **5** | **4** |
| ***Chlamydia pneumoniae*** | **1** | **-** | **1** | **-** |
| ***Legionella pneumophila*** | **-** | **-** | **-** | **-** |
| **Rhinovirus or enterovirus** | **92** | **86** | **59** | **38** |
| **Any influenza A virus** | **24** | **23** | **19** | **17** |
| Influenza A virus | 23 | 21 | 19 | 15 |
| Influenza A subtype H1 | - | - | - | - |
| Influenza A subtype H1N1/2009 | 12 | 12 | 6 | 6 |
| Influenza A subtype H3 | 12 | 11 | 9 | 9 |
| **Influenza B virus** | **9** | **9** | **7** | **6** |
| **Adenovirus** | **21** | **15** | **16** | **10** |
| **Respiratory syncytial virus A/B** | **14** | **14** | **10** | **8** |
| **Any parainfluenza virus** | **10** | **9** | **6** | **3** |
| Parainfluenza virus 1 | 1 | 1 | 1 | 1 |
| Parainfluenza virus 2 | 3 | 3 | 2 | 1 |
| Parainfluenza virus 3 | 4 | 4 | 2 | 1 |
| Parainfluenza virus 4 | 2 | 1 | 1 | - |
| **Human metapneumovirus A/B** | **8** | **6** | **6** | **7** |
| **Any coronavirus (excl. SARS-CoV-2)** | **6** | **6** | **3** | **3** |
| Coronavirus 229E | 1 | 1 | - | - |
| Coronavirus HKU1 | 2 | 2 | 2 | 1 |
| Coronavirus NL63 | 1 | 1 | - | - |
| Coronavirus OC43 | 2 | 2 | 1 | 2 |
| **SARS-CoV-2** | **5** | **5** | **3** | **1** |
| **Bocavirus** | **4** | **3** | **1** | **-** |

Abbreviations: NPS, nasopharyngeal swab

**Supplementary Figure 1: The samples and results** **by age.**





Stacked columns represent all 302 participants in each age group. Columns for “no sample” represent participants who refused or failed to provide a sample.

**Supplementary Figure 2.** **Cycle threshold values of all samples**





Cycle threshold values are shown for all detections, whether in 1, 2, or 3 samples. Cycle threshold values inversely correlate with pathogen load. Abbreviations: NPS, nasopharyngeal swab; IQR, interquartile range.

**Supplementary Figure 3.** **Pairwise comparisons of cycle threshold values**





The figure presents pairwise comparisons of cycle threshold values that were available for both compared samples (i.e., a positive test result). A paired t-test was used. For the influenza A virus, cycle threshold values from the generic influenza A virus assay were used, and participants who only had a positive detection in a subtype were excluded. Bocavirus and *Chlamydia pneumoniae* are not shown, as there were no detections in NPS with concordant detection in either saliva or mouthwash. Cycle threshold values inversely correlate with pathogen load. Abbreviations: NPS, nasopharyngeal swab; mouthw., mouthwash; IQR, interquartile range.

**Systematic review**

We conducted a systematic review on saliva samples compared to nasopharyngeal and oropharyngeal samples. Database-search was conducted on September 4^th^, 2024.

P: Under 18 years of age, respiratory tract infection or contact tracing

I: PCR of respiratory pathogen in saliva-based sample (saliva, oral/buccal swab or lollipop, gargle, mouthwash, oral rinse – excluding oropharyngeal swab, sputum, nasopharyngeal/nasal sample, pooled samples)

C: PCR of respiratory pathogen in nasopharyngeal or oropharyngeal sample

O: Sensitivity

Scopus: ( TITLE-ABS-KEY ( child* OR adolescen* OR pediatric* OR paediatric* OR neonate* OR newborn* OR infant* OR minor* OR toddler* OR teen* OR juvenile* ) AND TITLE-ABS-KEY ( saliva* OR spit OR spitting OR lollisponge OR lollipop* OR buccal* OR "mouth swab*" OR "oral swab*" OR gargle OR moutwash OR rins* OR "oral fluid*" OR "Respiratory Aerosols and Droplets" ) AND TITLE-ABS-KEY ( respiratory OR lung* OR bronchi* OR covid-19 OR "Common Cold" OR laryngitis OR pharyngitis OR pneumoni* OR rhinitis OR "Whooping Cough" OR coronavirus* OR flu OR flu-like OR adenov* OR bocav* OR hbov OR pertussi* OR sars-cov-2 OR oc43 OR hku1 OR nl63 OR 229e OR influenza* OR a-influenz* OR b-influenz* OR h1n1 OR legionell* OR pneumophil* OR metapneumo* OR hmpv OR mycoplasm* OR parainfluenzav* OR pin* OR rhinov* OR enterov* OR picornav* OR rsv ) AND TITLE-ABS-KEY ( "polymerase chain reaction" OR pcr ) )

Pubmed: (((("Adolescent"[Mesh] OR "Child"[Mesh] OR "Infant"[Mesh]) OR (Child*[Title/Abstract] OR adolescen*[Title/Abstract] OR pediatric*[Title/Abstract] OR paediatric*[Title/Abstract] OR neonate*[Title/Abstract] OR newborn*[Title/Abstract] OR infant*[Title/Abstract] OR minor*[Title/Abstract] OR toddler*[Title/Abstract] OR teen*[Title/Abstract] OR juvenile*[Title/Abstract])) AND (("Saliva"[Mesh] OR "Respiratory Aerosols and Droplets"[Mesh]) OR (saliva*[Title/Abstract] OR spit[Title/Abstract] OR spitting[Title/Abstract] OR LolliSponge[Title/Abstract] OR Lollipop*[Title/Abstract] OR buccal*[Title/Abstract] OR "mouth swab*"[Title/Abstract] OR "oral swab*"[Title/Abstract] OR Gargle[Title/Abstract] OR Moutwash[Title/Abstract] OR rins*[Title/Abstract] OR "oral fluid*"[Title/Abstract]))) AND (("Respiratory Tract Infections"[Mesh] OR "Influenza A virus"[Mesh] OR "Influenza B virus"[Mesh] OR "Coronavirus"[Mesh] OR "COVID-19 Testing"[Mesh] OR "Coronavirus Infections"[Mesh] OR "Metapneumovirus"[Mesh] OR "Picornaviridae"[Mesh] OR "Respiratory Syncytial Virus, Human"[Mesh] OR "Mycoplasma pneumoniae"[Mesh]) OR (respiratory[Title/Abstract] OR lung*[Title/Abstract] OR bronchi*[Title/Abstract] OR covid-19[Title/Abstract] OR "Common Cold"[Title/Abstract] OR Laryngitis[Title/Abstract] OR Pharyngitis[Title/Abstract] OR Pneumoni*[Title/Abstract] OR Rhinitis[Title/Abstract] OR "Whooping Cough"[Title/Abstract] OR Coronavirus*[Title/Abstract] OR flu[Title/Abstract] OR flu-like[Title/Abstract] OR Adenov*[Title/Abstract] OR Bocav*[Title/Abstract] OR HBoV[Title/Abstract] OR Pertussi*[Title/Abstract] OR SARS-CoV-2[Title/Abstract] OR OC43[Title/Abstract] OR HKU1[Title/Abstract] OR NL63[Title/Abstract] OR 229E[Title/Abstract] OR Influenza*[Title/Abstract] OR A-influenz*[Title/Abstract] OR B-influenz*[Title/Abstract] OR H1N1[Title/Abstract] OR Legionell*[Title/Abstract] OR pneumophil*[Title/Abstract] OR Metapneumo*[Title/Abstract] OR HMPV[Title/Abstract] OR Mycoplasm*[Title/Abstract] OR Parainfluenzav*[Title/Abstract] OR Rhinov*[Title/Abstract] OR Enterov*[Title/Abstract] OR picornav*[Title/Abstract] OR RSV[Title/Abstract]))) AND (("Polymerase Chain Reaction"[Mesh]) OR ("polymerase chain reaction"[Title/Abstract] OR PCR[Title/Abstract]))

**Supplementary Figure 4. Studies screened for the systematic review**

Studies identified **(n = 1087)**

Scopus (n = 724)

PubMed (n = 358)

Citation searching (n = 5)

Studies screened **(n = 781)**

Duplicates removed **(n = 306)**

Studies excluded **(n = 628)**

Studies assessed for eligibility **(n = 153)**

Studies excluded **(n = 118)**

Duplicate (n = 1)

Wrong setting / study design (n = 50)

Adult population (n = 26)

Children not separated from adults (n = 39)

Text not in English (n = 2)

Studies included **(n = 35)**

Studies on multiplex PCR (n = 3)

Other studies on multiple pathogens (n = 3)

Studies on influenza (n = 1)

Studies on SARS-CoV-2 (n = 25)

Other studies on single pathogens (n = 3)

**Supplementary Table 4. Studies included in the systematic review**

| **Study** | **Country & year** | **n** | **Population** | **Age range** | **Age (median / mean)** | **Saliva sample type** | **Comparator** | **Pathogens** |
| --- | --- | --- | --- | --- | --- | --- | --- | --- |
| **Studies on multiplex PCR** | | | | | | | | |
| de Koff 2021^1^ | Netherlands  2020 | 57 | Suspected COVID-19 | 0–18 | 2.1 [IQR 1.1–3.5] | Oral swab AND drooling AND sponge (ORACOL) | NPS and OPS | Multiplex (RV, InfA, InfB, RSV, HMPV, AdV, BoV, CoV, PIN, *B. pertussis*, *M. pneumoniae*) |
| Lown 2022^2^ | UK  2020 | 11 | Sore throat or asymptomatic control | 6–15 | 9 [IQR 5.5–12.5] | Drooling AND sponge | OPS | Multiplex (AdV, SARS-CoV-2, InfA, InfB, HMPV, PIN, RV, RSV, streptococcus A/B/C/F/G) |
| Buonsenso 2023^3^ | Italy  2021–22 | 83 | Acute RTI | 0–17 | 1.0 [IQR 2.5] | Sponge (LolliSponge) | NPS/OPS | Multiplex (AdV, BoV, CoV-229E, CoV-HKU1, CoV-NL63, CoV-OC43, SARS-CoV-2, InfA, InfA-H1, InfA-H1N1, InfA-H3, InfB, HMPV, PIN1-4, RSV, RV/EV, *B. pertussis*, *L. pneumophila*, *M. pneumoniae*) |
| **Studies on multiple pathogens (other than multiplex PCR)** | | | | | | | | |
| von Linstow 2006^4^ | Denmark | 44 | Hospitalization for HMPV or RSV | 0–3 |  | NS | NPS | HMPV, RSV |
| Robinson 2008^5^ | Canada  2006–07 | 105 | Suspected LRTI and pathogen detected in NPS | 0–8.1 | 0.4 | Sponge (ORACOL) | NPS | AdV, InfA, InfB, HMPV, PIN, RSV |
| Woodall 2021^6^ | UK | 106 | RTI |  | 2.6 [IQR 1.5–5] | Oral swab (nurse-collected AND parent-collected) | NPS | 29 viruses and 13 bacteria (e.g., AdV, BoV, *B. pertussis*, CoV-NL63, InfB, HMPV, PIN1-4, RV, EV) |
| **Studies on influenza virus** | | | | | | | | |
| Kaku 2023^7^ | Japan  2019–20 | 37 | Influenza-like illness | <16 | NS | Gargle | NPS | Influenza viruses A and B |
| **Studies on SARS-CoV-2** | | | | | | | | |
| Alenquer 2022^8^ | Portugal  2020–21 | 85 | Hospitalization for any reason | 0–10 | 3.8 [SD 3.4] | Drooling or aspiration (< 1 y.) | NPS | SARS-CoV-2 |
| Al Suwaidi 2021^9^ | UAE  2020 | 485 | COVID-19 screening | 3–18 | 10.8 [SD 3.9] | Drooling | NPS | SARS-CoV-2 |
| Ana Laura 2021^10^ | Mexico  2020 | 156 | Inpatients' COVID-19 screening | 5–18 | 11 [IQR 7–14] | Drooling | NPS/OPS | SARS-CoV-2 |
| Borghi 2021^11^ | Italy | 109 | Symptomatic and asymptomatic | 0–17 | NS | Sponge (SalivaDirect) | NPS | SARS-CoV-2 |
| Calvet 2023^12^ | Brazil  2022 | 111 | COVID-19 suspicion / contact tracing | 3–17 | 9.7 [IQR 7.2–13.4] | Drooling | NPS/OPS | SARS-CoV-2 |
| Chong 2021^13^ | Singapore  2020 | 18 | NPS-confirmed COVID-19 | NS | 6.6 [IQR 1.8–11.1] | Drooling/aspiration | NPS | SARS-CoV-2 |
| Delaunay-Moisan 2022^14^ | France  2020–21 | 272 | Hospitalization for any reason / COVID-19 contact tracing | 0–17 | NS | Drooling or aspiration (< 3 y.) | NPS | SARS-CoV-2 |
| Devina 2023^15^ | Indonesia  2020 | 36 | NPS-confirmed COVID-19 | 11–12 | NS | Drooling | NPS | SARS-CoV-2 |
| Diani 2023^16^ | Italy  2020 | 256 | Hospitalization for any reason | NS | 4.2 | Oral swab | NPS | SARS-CoV-2 |
| Fougère 2021^17^ | Switzerland  2020 | 397 | COVID-19 suspicion | 0–18 | 12.7 [SD 3.8] | Drooling | NPS | SARS-CoV-2 |
| Gaur 2021^18^ | India  2020 | 13 | NPS-confirmed COVID-19 | 13–16 | 15 | Drooling AND oral swap | NPS | SARS-CoV-2 |
| Han 2020^19^ | South Korea  2020 | 11 | NPS-confirmed COVID-19 | 0–16 | 6.5 | NS | NPS/OPS | SARS-CoV-2 |
| Huber 2021^20^ | Switzerland  2020–21 | 170 | COVID-19 testing for any reason | 5–17 | 13 | Drooling | NPS | SARS-CoV-2 |
| Isabel 2023^21^ | Canada  2021 | 82 | COVID-19 suspicion / contact tracing | 4–18 | NS | Gargle | NPS | SARS-CoV-2 |
| Kam 2020^22^ | Singapore  2020 | 11 | NPS-confirmed COVID-19 | 0–12 | NS | Oral swap | NPS | SARS-CoV-2 |
| Kim 2024^23^ | Korea  2021 | 251 | COVID-19 testing for any reason | 0– | NS | Drooling or swab/aspiration (< 2 y.) | NPS or NPA | SARS-CoV-2 |
| McBride 2024^24^ | USA  2022–23 | 99 | Suspected COVID-19 | NS | NS | Oral swab | NPS | SARS-CoV-2 |
| Monzani 2022^25^ | Italy  2020–21 | 35 | NPS-confirmed COVID-19 | 0–15 | 8 [IQR 2–13] | Aspiration | NPS | SARS-CoV-2 |
| Moraleda 2022^26^ | Spain  2021 | 1174 | Suspected COVID-19 | 0–18 | 3.8 [IQR 1.7–9.0] | Oral swab | NPS | SARS-CoV-2 |
| Oliver 2021^27^ | Australia  2020 | 176 | COVID-19 testing for any reason | –10 | NS | Drooling or oral swab (< 5 y.) | NPS/OPS | SARS-CoV-2 |
| Pinninti 2021^28^ | USA  2020 | 63 | NPS-confirmed COVID-19 | 0–21 | 9.8 [SD 6.6] | Oral swab | NPS | SARS-CoV-2 |
| Salu 2022^29^ | Nigeria  2021 | 22 | COVID-19 testing for any reason | –20 | NS | Drooling | NPS | SARS-CoV-2 |
| Trobajo-Sanmartín 2021^30^ | Spain  2020 | 103 | Suspected COVID-19 | 0–14 | NS | Drooling | NPS | SARS-CoV-2 |
| Vos 2022^31^ | USA  2020–21 | 142 | COVID-19 testing for any reason | 5–19 | NS | Drooling | NPS | SARS-CoV-2 |
| Yee 2021^32^ | USA  2020 | 43 | COVID-19 testing for any reason | 4–18 | 12 | Drooling | NPS | SARS-CoV-2 |
| **Studies on other single pathogens** | | | | | | | | |
| Martin 2009^33^ | USA  2007–08 | 149 | Asymptomatic and retested at the onset of RTI | 2–11 | NS | Schirmer test filter paper | NPS | BoV |
| DeMuri 2020^34^ | USA  2019 | 20 | Antigen test confirmed GAS-tonsillitis | 6–15 | 9.7 | Oral swab | OPS | GAS |
| Hashavya 2020^35^ | Israel  2017–18 | 100 | Acute tonsillitis | NS | 8.4 [SD 2.6] | Drooling | OPS | GAS |

Abbreviations: RTI, respiratory tract infection; GAS, Group A streptococcus; BoV, bocavirus; RV, rhinovirus; InfA, influenza A virus; InfB, influenza B virus; RSV, respiratory syncytial virus; HMPV, human metapneumovirus; AdV, adenovirus; CoV, coronavirus; PIN, paraninfluena virus; EV, enterovirus; NPS, nasopharyngeal swab; NPA, nasopharyngeal aspirate; OPS, oropharyngeal swab; NS, not specified.

**References**

1. de Koff EM, Euser SM, Badoux P, et al. Respiratory pathogen detection in dhildren: saliva as a diagnostic specimen. *Pediatr Infect Dis J*. 2021;40(9):e351-e353.

2. Lown M, Miles EA, Fisk HL, et al. Self-sampling to identify pathogens and inflammatory markers in patients with acute sore throat: Feasibility study. *Front Immunol*. 2022;13:1016181.

3. Buonsenso D, Valentini P, Mariani F, et al. Comparison between nasopharyngeal and saliva samples for the detection of respiratory viruses in children with acute lower respiratory tract infections: a pilot study. *Children (Basel)*. 2023;10(5).

4. von Linstow ML, Eugen-Olsen J, Koch A, Winther TN, Westh H, Hogh B. Excretion patterns of human metapneumovirus and respiratory syncytial virus among young children. *Eur J Med Res*. 2006;11(8):329-335.

5. Robinson JL, Lee BE, Kothapalli S, Craig WR, Fox JD. Use of throat swab or saliva specimens for detection of respiratory viruses in children. *Clin Infect Dis*. 2008;46(7):e61-4.

6. Woodall CA, Thornton HV, Anderson EC, et al. Prospective study of the performance of parent-collected nasal and saliva swab samples, compared with nurse-collected swab samples, for the molecular detection of respiratory microorganisms. *Microbiol Spectr*. 2021;9(3):e0016421.

7. Kaku N, Urabe T, Iida T, et al. Gargle sample is an effective option in a novel fully automated molecular point-of-care test for influenza: a multicenter study. *Virol J*. 2023;20(1):41.

8. Alenquer M, Milheiro Silva T, Akpogheneta O, et al. Saliva molecular testing bypassing RNA extraction is suitable for monitoring and diagnosing SARS-CoV-2 infection in children. PLoS One. 2022;17(6):e0268388.

9. Al Suwaidi H, Senok A, Varghese R, et al. Saliva for molecular detection of SARS-CoV-2 in school-age children. *Clin Microbiol Infect*. 2021;27(9):1330-1335.

10. Ana Laura GO, Abraham Josué NR, Briceida LM, et al. Sensitivity of the molecular test in saliva for detection of COVID-19 in pediatric patients with concurrent conditions. *Front Pediatr*. 2021;9:642781.

11. Borghi E, Massa V, Carmagnola D, et al. Saliva sampling for chasing SARS-CoV-2: a game-changing strategy. *Pharmacol Res*. 2021;165:105380.

12. Calvet G, Ogrzewalska M, Tassinari W, et al. Accuracy of saliva for SARS-CoV-2 detection in outpatients and their household contacts during the circulation of the Omicron variant of concern. *BMC Infect Dis*. 2023;23(1):295.

13. Chong CY, Kam KQ, Li J, et al. Saliva is not a useful diagnostic specimen in children with coronavirus disease 2019 (COVID-19). *Clin Infect Dis*. 2021;73(9):E3144-E3145.

14. Delaunay-Moisan A, Guilleminot T, Semeraro M, et al. Saliva for molecular detection of SARS-CoV-2 in pre-school and school-age children. *Environ Microbiol*. 2022;24(10):4725-4737.

15. Devina C, Nasution BB, Kusumawati RL, Daulay RS, Trisnawati Y, Lubis IND. Sensitivity of nasopharyngeal swab and saliva specimens in the detection of SARS-CoV-2 virus among boarding school girls. *IJID Reg*. 2023;8(Suppl):S13-7.

16. Diani E, Silvagni D, Lotti V, et al. Evaluation of saliva and nasopharyngeal swab sampling for genomic detection of SARS-CoV-2 in children accessing a pediatric emergency department during the second pandemic wave. *Front Microbiol*. 2023;14:1163438.

17. Fougère Y, Schwob JM, Miauton A, et al. Performance of RT-PCR on saliva specimens compared with nasopharyngeal swabs for the detection of SARS-CoV-2 in children: a prospective comparative clinical trial. *Pediatr Infect Dis J*. 2021;40(8):e300-e304.

18. Gaur R, Verma DK, Mohindra R, et al. Buccal swabs as non-invasive specimens for detection of severe acute respiratory syndrome coronavirus-2. *J Int Med Res*. 2021;49(5).

19. Han MS, Seong MW, Kim N, et al. Viral RNA load in mildly symptomatic and asymptomatic children with COVID-19, Seoul, South Korea. *Emerg Infect Dis*. 2020;26(10):2497-2499.

20. Huber M, Schreiber PW, Scheier T, et al. High efficacy of saliva in detecting SARS-CoV-2 by RT-PCR in adults and children. *Microorganisms*. 2021;9(3).

21. Isabel S, Cohen-Silver J, Jung H, et al. Swish and gargle saliva sampling is a patient-friendly and comparable alternative to nasopharyngeal swabs to detect SARS-CoV-2 in outpatient settings for adults and children. *Microbiol Spectr*. 2023;11(6).

22. Kam KQ, Yung CF, Maiwald M, et al. Clinical utility of buccal swabs for severe acute respiratory syndrome coronavirus 2 detection in coronavirus disease 2019-infected children. *J Pediatric Infect Dis Soc*. 2020;9(3):370-372.

23. Kim MJ, Park PG, Hwang SJ, et al. Saliva-based proteinase K method: a rapid and reliable diagnostic tool for the detection of SARS-COV-2 in children. *J Med Virol*. 2024;96(1):e29361.

24. McBride JA, DeMuri G, Nelson C, et al. Performance characteristics of “lollipop” swabs for the diagnosis of infection with SARS-CoV-2. *Diagn Microbiol Infect Dis*. 2024;110(1).

25. Monzani A, Borgogna C, Ferrante D, et al. #Stayathome if you have a cold: high SARS-CoV-2 salivary viral loads in pediatricp patients with nasopharyngeal symptoms. *Viruses*. 2022;15(1).

26. Moraleda C, Domínguez-Rodríguez S, Mesa JM, et al. Oral saliva swab reverse transcription PCR for Covid-19 in the paediatric population. *Arch Dis Child*. 2022;107(11):1051-1058.

27. Oliver J, Tosif S, Lee LY, et al. Adding saliva testing to oropharyngeal and deep nasal swab testing increases PCR detection of SARS-CoV-2 in primary care and children. *Med J Aust*. 2021;215(6):273-278.

28. Pinninti SG, Pati S, Poole C, et al. Virological characteristics of hospitalized children with SARS-CoV-2 infection. *Pediatrics*. 2021;147(5).

29. Salu OB, Akase IE, Anyanwu RA, et al. Saliva sample for detection of SARS-CoV-2: a possible alternative for mass testing. *PLoS ONE*. 2022;17(9 September).

30. Trobajo-Sanmartín C, Adelantado M, Navascués A, et al. Self-collection of saliva specimens as a suitable alternative to nasopharyngeal swabs for the diagnosis of SARS-CoV-2 by RT-qPCR. *J Clin Med*. 2021;10(2):1-9.

31. Vos MB, Gonzalez MD, Stone C, et al. Comparison of mid-turbinate nasal swabs, saliva, and nasopharyngeal swabs for SARS-CoV-2 reverse transcription-polymerase chain reaction testing in pediatric outpatients. *Arch Pathol Lab Med*. 2022;146(9):1056-1061.

33. Yee R, Truong TT, Pannaraj PS, et al. Saliva is a promising alternative specimen for the detection of SARS-CoV-2 in children and adults. *J Clin Microbiol*. 2021;59(2).

33. Martin ET, Taylor J, Kuypers J, et al. Detection of bocavirus in saliva of children with and without respiratory illness. *J Clin Microbiol*. 2009;47(12):4131-4132.

34. DeMuri G, Wald ER. Detection of group A streptococcus in the saliva of children presenting with pharyngitis using the cobas Liat PCR system. *Clin Pediatr (Phila)*. 2020;59(9-10):856-858.

35. Hashavya S, Pines N, Gayego A, Schechter A, Gross I, Moses A. The use of bacterial DNA from saliva for the detection of GAS pharyngitis. *J Oral Microbiol*. 2020;12(1):1771065.
